# microRNA-216b enhances cisplatin-induced apoptosis in osteosarcoma MG63 and SaOS-2 cells by binding to JMJD2C and regulating the HIF1α/HES1 signaling axis

**DOI:** 10.1186/s13046-020-01670-3

**Published:** 2020-09-24

**Authors:** Dong Yang, Tianyang Xu, Lin Fan, Kaiyuan Liu, Guodong Li

**Affiliations:** Department of Orthopedics, Shanghai Tenth People’s Hospital, No. 301, Yanchang Road, Shanghai, 200072 P.R. China

**Keywords:** microRNA-216b, JMJD2C, HIF1α, HES1, Osteosarcoma, Cisplatin

## Abstract

**Background:**

Although cisplatin-based chemotherapy represents the standard regimen for osteosarcoma (OS), OS patients often exhibit treatment failure and poor prognosis due to chemoresistance to cisplatin. Emerging research has highlighted the tumor suppressive properties of microRNAs (miRNAs or miRs) in various human cancers via the inhibition of the histone demethylase jumonji domain containing protein 2C (JMJD2C). As a coactivator for hypoxia-inducible factor 1α (HIF1α), JMJD2C targets hairy and enhancer of split-1 (HES1) gene. Hence, the current study aimed to elucidate the role of miR-216b in OS cell cisplatin resistance to identify the underlying mechanism of miR-216b regulating the JMJD2C//HIF1α/HES1 signaling.

**Methods:**

Tumor and paracancerous tissues were collected from OS patients to determine the expression patterns of miR-216b and JMJD2C. After ectopic expression and knockdown experiments in the OS cells, CCK-8 assay and flow cytometry were employed to determine cell viability and apoptosis. The interaction of miR-216b, JMJD2C, HIF1α and HES1 was subsequently determined by dual luciferase reporter, co-immunoprecipitation (IP) and ChIP-qPCR assays. In vivo experiments were conducted to further verify the role of the miR-216b in the resistance of OS cells to cisplatin.

**Results:**

miR-216b expression was reduced in the OS tissues, as well as the MG63 and SaOS-2 cells. Heightened miR-216b expression was found to be positively correlated with patient survival, and miR-216b further enhanced cisplatin-induced apoptosis of MG63 and SaOS-2 cells. Mechanistically, miR-216b inhibited JMJD2C expression by binding to its 3’UTR. Through interaction with HIF1α, JMJD2C removed the H3K9 methylation modification at the HES1 promoter region, leading to upregulation of HES1 in vitro. Furthermore, miR-216b was observed to increase the tumor growth in nude mice in the presence of cisplatin treatment. HES1 overexpression weakened the effects of miR-216b in MG63 and SaOS-2 cells and in nude mouse xenografts.

**Conclusion:**

Overall, miR-216b enhanced the sensitivity of OS cells to cisplatin via downregulation of the JMJD2C/HIF1α/HES1 signaling axis, highlighting the capacity of miR-216b as an adjunct to cisplatin chemotherapy in the treatment of OS.

## Background

Osteosarcoma (OS) represents one of the most common primary bone malignancies, predominantly affecting children and adolescents [1]. In addition to the highest incidence of OS in adolescence, a second incidence peak has been reported in the elderly, a high-risk population group [2]. The incidence of OS is estimated as ‘4’ for the 0–14 years age range and ‘5’ for 15–19 years age range per million people worldwide [3]. Moreover, the 5-year survival rate worldwide for OS remains to be largely appalling, with studies estimating a survival rate between 40 and 70% [3, 4], a statistic which has failed to improve for decades [5]. Young age, first primary tumor, localized stage, low grade, and surgical treatment are factors that have all been positively linked with overall survival in OS [6]. Currently, a combination of surgical intervention as well as chemotherapy is regarded as the first line treatment for OS. However, it is well-documented that the advent of chemotherapy resistance is a big obstacle in the treatment of OS [7–10], which may, at least partly, contribute to the unfavorable outcomes seen in OS patients. Hence, the current study set out to identify the potential mechanism by which chemotherapy drug resistance occurs during the course of OS treatment.

microRNAs (miRNAs or miRs) are known to possess the ability to regulate cell proliferation and apoptosis, and as a result, are implicated in a wide variety of human cancers as oncogenes or tumor suppressors [11]. MiRNAs refer to small non-coding RNA molecules, which serve as regulators of protein expression via the degradation of targeted mRNA or blockade of protein translation [12]. More notably, one such miRNA, miR-216b has been recently shown to potentially exert a tumor inhibitor function in OS by targeting FoxM1 as it inhibits OS cell proliferation, migration, and invasion [13]. Moreover, the over-expression of miR-216b has also been previously demonstrated to significantly enhance the sensitivity of non-small cell lung cancer cells to cisplatin-induced apoptosis by targeting c-Jun [14]. In addition, miR-216b can diminish cell proliferation, while promoting the sensitivity of colorectal cancer cells to oxaliplation by suppressing PDZ-binding kinase [15]. These findings lead the authors to hypothesize that miR-216b may confer a similar role in OS to affect the chemosensitivity of OS cells.

Histone demethylase jumonji C domain-containing 2C (JMJD2C) is known to demonstrate regulatory potentials in the epigenetic mechanism in malignant diseases, particularly in regard to moderating the influence on the promoter activity of target genes which are strongly associated with tumor development [16]. Prior evidence has proposed that JMJD2C is highly-expressed in OS, and even confers a regulatory role in the context of OS [17]. Moreover, JMJD2C has also been shown to serve as a co-activator for hypoxia-inducible factor 1 (HIF1α) for cancer progression [18]. Meanwhile, as a widely-documented regulator of cellular metabolism, HIF1α performs essential functions in the survival and differentiation of mesenchymal stem cells [19]. HIF1α has been reported to be highly-expressed in OS [20], and to further function to activate downstream genes through its transcriptional activation to promote OS [21]. More importantly, HIF1α has been reported to induce drug resistance through the hairy and enhancer of split-1 gene (HES1) in breast cancer [22]. HES1 represents a critical factor for the maintenance of stem cells, quiescent cells or cancer cells, and has also been demonstrated to elicit drug resistance and metastasis of tumor cells [23]. Furthermore, HES1 has been reported to be highly-active in OS [24], with studies suggesting that HES1 promotes the critical phenomenon of chemotherapy resistance in cancer [25, 26]. In order to effectively inhibit HES1 to reduce chemotherapy resistance, one method is to modify H3K9 methylation at its gene promoter [27]. Hence, the current study set out to examine whether miR-216b inhibits cancer growth in OS by reducing the levels of JMJD2C and HES1. We further aimed to determine the involvement of HIF1α and H3K9 methylation modification in the underlying mechanism associated with the effects of miR-216b.

## Materials and methods

### Ethics statement

The current study was performed with the approval of the Ethics committee of Shanghai Tenth People’s Hospital (approval number: 2010–0017) and in strict accordance with the *Declaration of Helsinki*. Signed informed consents were obtained from all participants or their guardians prior to the study. Animal experiments were performed according to a strictly designed protocol, in accordance with the Guide for the Care and Use of Laboratory Animals published by the US National Institutes of Health. All efforts were made to ensure minimal suffering of the animal included in the study.

### Sample collection from OS patients

Cancer tissues and paracancerous tissues were collected from a total of 60 OS patients (aged 10–58 years, with an average age of 21.63 ± 9.70 years old) at the Shanghai Tenth People’s Hospital from April 2010 to April 2013. All included patients had not undergone chemotherapy or radiotherapy prior to specimen collection. Patient follow-up was conducted over a period of 60 months. The time from specimen collection to tumor recurrence or death (overall survival) was recorded. The last follow-up date was recorded if no tumor recurrence or death occurred. All included patients received the same treatment regimen at the Shanghai Tenth People’s Hospital, with no metastasis occurring during the follow-up period.

### Cell transfection

Human osteosarcoma cell lines U2OS, HOS, SaOS-2, MG-63 and human embryonic immortalized osteoblasts hFOB1.19 cells (American Type Culture Collection, Manassas, VA, USA) were cultured in Dulbecco’s modified Eagle’s medium (DMEM; HyClone, Logan, UT USA) supplemented with 10% fetal bovine serum, 100 μg/mL streptomycin, and 100 U/mL penicillin at 37 °C with 5% CO_2_ [28].

The MG63 and SaOS-2 cells were subsequently transfected with agomir negative control (NC), miR-216b agomir, agomir NC + over-expression (oe)-NC, miR-216b agomir + oe-NC, miR-216b agomir + oe-JMJD2C, miR-216b agomir + oe-HES1, oe-NC + lentivirus (LV)-short hairpin RNA (sh)NC, oe-JMJD2C + LV-shNC, oe-JMJD2C + LV-shHIF1α, LV-shJMJD2C + oe-NC, and LV-shJMJD2C + oe-HIF1α. The cDNA constructs (pcDNA3.1-NC, pcDNA3.1-JMJD2C, pcDNA3.1-HIF1α and pcDNA3.1-HES1) were all constructed by HanBio Biotechnology Co., Ltd. (Shanghai, China). Meanwhile, agomir NC (5′-UUCUCCGAACGUGUCACGUTT-3′), miR-216b agomir (5′-AAAUCUCUGCAGGCAAAUGUGA-3′), LV-shNC, LV-shHIF1α, LV-shJMJD2C were all designed and constructed by GenePharma (Shanghai, China). Transfection was performed using Lipofectamine 2000 reagents (Invitrogen, Carlsbad, CA, USA) in accordance with the manufactures’ instructions. JMJD2C (gene ID: 23081), HIF1 (gene ID: 3091) and HES1 (gene ID: 3280) were identified by the National Center for Biotechnology Information (NCBI) database.

### RNA quantitation by reverse transcription quantitative polymerase chain reaction (RT-qPCR)

Total RNA content was extracted from the fresh tissues using RNeasy Mini kits (Qiagen, Valencia, CA, USA). For mRNA detection, the extracted total RNA was reverse transcribed into complementary DNA (cDNA) with the help of reverse transcription kits (RR047A, Takara, Japan). For miRNA detection, cDNA was obtained using miRNA First Strand cDNA Synthesis (Tailing Reaction) kits (B532451–0020, Sangon Biotech Co., Ltd., Shanghai, China). The samples were then loaded using a SYBR® Premix Ex TaqTM II (Perfect Real Time) kit (DRR081, Takara, Japan). RT-qPCR was performed on an ABI 7500 instrument (Applied Biosystems, Foster City, CA, USA), and each sample was evaluated in triplicate. The universal negative primers for miRNA, while the internal reference U6 were provided by miRNA First Strand cDNA Synthesis (Tailing Reaction) kit. The remaining primers were provided by Sangon Biotech Co., Ltd. (Shanghai, China). The primer sequences are depicted in Table 1. The Ct value of each target gene was recorded and normalized to an internal reference, namely, β-actin or U6. Relative expression of all target genes was calculated by means of relative quantification (2^-ΔΔCt^ method) [29].

**Table 1:** Primer sequences for RT-qPCR

Target	Primer sequence (5′-3′)
miR-216b	Forward: 5′-GCCGCGCTAAAGTGCTTATAGTG-3’
HIF-1α	Reverse: 5′-CACCAGGGTCCGAGGT-3’
	Forward: 5′-CAGAAGATACAAGTAGCCTC-3’
β-actin	Reverse: 5′-CTGCTGGAATACTGTAACTG-3’
	Forward: 5′-GCGAGAAGATGACCCAGGATC-3’
	Reverse: 5′-CCAGTGGTACGGCCAGAGG-3’
U6	Forward: 5′-ACGCTTCACGAATTTGCGTGTC-3’
	Reverse: 5′-GCTTCGGCAGCACATATACTAAAAT-3’

### Protein expression determined by western blot analysis

Total protein content was extracted from the tissues or cells using radioimmunoprecipitation assay (RIPA) lysis buffer containing phenylmethylsulphonyl fluoride (PMSF) on ice for 30 min, followed by centrifugation at 8000 g and 4 °C for 10 min. The supernatant was then collected for protein concentration determination using a bicinchoninic acid (BCA) protein assay kit (Thermo Fisher Scientific, Rockford, IL, USA, #23250). Protein (50 μg) was subsequently dissolved in 2 × sodium dodecyl sulfate (SDS) loading buffer, boiled at 100 °C for 5 min, and then separated using SDS-polyacrylamide gel electrophoresis (PAGE). The protein was subsequently transferred onto polyvinylidene fluoride (PVDF) membranes, which were blocked by 5% skim milk at room temperature for 1 h and incubated with primary rabbit antibodies against JMJD2C (ab85454, dilution ratio of 1:1000), HIF1α (ab51608, dilution ratio of 1:500), HES1 (ab71559, dilution ratio of 1:500), Ki67 (ab92742, dilution ratio of 1:5000), B-cell lymphoma 2 (Bcl-2) (ab32124, dilution ratio of 1:1000), and β-actin (ab227387, dilution ratio of 1:5000) overnight at 4 °C. After three Tris-buffered saline Tween-20 (TBST) washes (10 min per wash), the membranes were then incubated with horseradish peroxidase (HRP)-labeled secondary goat anti-rabbit immunoglobulin G (IgG) H&L (ab97051, dilution ratio of 1:2000) for 1 h. All the aforementioned antibodies were purchased from Abcam Inc. (Cambridge, UK). The immunocomplexes on the membrane were visualized using enhanced chemiluminescence (ECL) reagents (BB-3501, Amersham, UK), with the band intensities quantified with the Bio-Rad Image Analysis System (Bio-Rad, Inc., Hercules, CA, USA), and Quantity One v4.6.2 software. The ratio of the gray value of the target band to β-actin was regarded as a reflection of the relative protein expression.

### Binding relationship determined by dual luciferase reporter gene assay

Wild type (wt) (pGL3-wt-JMJD2C-3’untranslated region [UTR]) or mutant (mut) (pGL3-mut-JMJD2C-3’UTR) reporter plasmids (GenePharma, Shanghai, China) were co-transfected with agomir NC or miR-216b agomir into MG63 and SaOS-2 cells. Following a 48 h period of transfection, the cells were collected and lysed. The subsequent procedures were performed with using a luciferase assay kit (K801–200, Biovision, Milpitas, CA, USA) following the manufacturer’s instructions on the luciferase reporter assay system (Promega, Madison, WI, USA). The relative luciferase activity was calculated based on the ratio of the luciferase activity of firefly luciferase to that of renilla luciferase. The sequences of wt-JMJD2C-3’UTR and mut-JMJD2C-3’UTR were 5′-GCAUGUAUGCUAAUGAGAUUU-3′ and 5′-GCUGUAAACGACGUCUCUAAA-3′, respectively [30].

### Binding of JMJD2C to HIF1α determined by co-immunoprecipitation (co-IP) assay

The cells were lysed for 30 min at 4 °C in RIPA buffer (Thermo Scientific, Waltham, MA, USA), and then centrifuged at 13,000 g for 30 min at 4 °C. The supernatant was subsequently collected and incubated with specific antibody at 4 °C overnight, followed by the addition of pierce protein A/G Magnetic Beads (#88803, Thermo Scientific) and 4 h of incubation by shaking at 4 °C. The beads were then centrifuged, washed three times, and mixed with the loading buffer, followed by protein evaluation using SDS-PAGE and immunoblotting [31].

### Quantity of the HES1 promoter determined by chromatin immunoprecipitation (ChIP)-qPCR

ChIP was performed using EZ-Magna ChIP A/G Chromatin Immunoprecipitation kits (17–371, Millipore, Billerica, MA, USA). Briefly, the cells were sonicated and centrifuged at 12,000 g for 10 min at 4 °C to remove insoluble precipitates. The cells were subsequently incubated with protein G agarose beads at 4 °C for 1 h, and centrifuged at 5000 g for a total of 1 min. Next, 10 μL (1%) of the supernatant was collected as the ‘Input’, while the remaining supernatant was divided into 2 portions and incubated with antibodies against H3K9me3 (ab8898, dilution ratio of 1:20, Abcam Inc., Cambridge, UK), H3K9me2 (ab1220, dilution ratio of 1:10, Abcam Inc., Cambridge, UK), H3 (ab213257, dilution ratio of 1:5, Abcam Inc., Cambridge, UK), and NC rabbit anti-human IgG (ab2410, dilution ratio of 1:25, Abcam Inc., Cambridge, UK) at 4 °C overnight. The precipitated protein-DNA complex was then incubated with protein G agarose beads for 1 h at 4 °C. After centrifugation at 5000 g for 1 min, the supernatant was discarded. The protein-DNA complex was fragmented overnight at 65 °C. The DNA fragment was recovered and used as an amplification template for RT-qPCR. The primers employed to detect the quantity of HES1 promoter were agomir NC (5′-UUCUCCGAACGUGUCACGUTT-3′) and miR-216b agomir (5′-AAAUCUCUGCAGGCAAAUGUGA-3′).

### Cell viability assays

Cell proliferation was measured using a cell counting kit-8 (CCK-8) assay (Kumamoto, Japan). MG63 and SaOS-2 cells were firstly seeded in 96-well plates. After 24 h of transfection, various concentrations of cisplatin (0, 0.2, 0.4, 0.8, 1.6 μM) were added to the cells and incubated for 72 h. The CCK-8 reagent (10 μL) was then added to each well and incubated for 1 h. Absorbance was finally measured at a wavelength of 450 nm using a microplate reader.

### Apoptosis assays

After 24 h of transfection, the cells were treated with 1 μM cisplatin [32] for 72 h. An apoptosis assay was subsequently performed using Annexin V-fluorescein isothiocyanate/propidium iodide (FITC/PI) kits (KeyGEN Biotech. Co., Ltd., Nanjing, China). The cells were subsequently observed and analyzed using a flow cytometer (FACSCalibur, BD Biosciences, Franklin Lakes, NY, USA). The experiment was performed in triplicate, with the respective average values obtained and recorded.

### Tumor xenograft in nude mice

A total of 30 BALB/c nude mice (aged 5–6 weeks old, weighing 15–18 g, acquired from the Shanghai Experimental Animal Center of Chinese Academy of Sciences) were randomly divided into the 5 following groups (6 mice per group): control group, Lv-oe-NC group, Lv-oe-NC + Cis group, Lv-oe-miR-216b + Lv-oe-NC + Cis group, and Lv-oe-miR-216b + Lv-oe-HES1 + Cis group. The lentiviruses carrying oe-NC, oe-miR-216b and oe-HES1 were purchased from Shanghai Gene Pharma Co., Ltd. (Shanghai, China). Following lentivirus infection, the stably-transfected MG63 cells were subcutaneously injected into the axillary region of nude mice at a density of 1 × 10^6^ cells. From the 7th day of inoculation, cisplatin (8 mg/kg, [33]) was intraperitoneally administered to the mice in the Lv-oe-NC + Cis, Lv-oe-miR-216b + Lv-oe-NC + Cis, and Lv-oe-miR-216b + Lv-oe-HES1 + Cis groups twice a week. On the 14th day, the mice were euthanized using carbon dioxide. The tumors were subsequently removed, after which the tumor volume was measured using the following formula: (a × b^2^)/2 [34], where a represented the length of the tumor and b was the width of the tumor. The volume of the tumor was calculated and a tumor growth curve was then plotted [35].

### Statistical analysis

Data analyses were processed using the SPSS 21.0 statistical software (IBM Corp. Armonk, NY, USA). Measurement data were expressed as mean ± standard deviation. Data with normal distribution and homogeneity of variance between two groups were compared using paired *t*-test (paired data) or unpaired *t*-test (unpaired data). Data comparisons between multiple groups were performed using one-way analysis of variance (ANOVA), followed by a Tukey’s test. Data comparison between groups at different time points was performed by repeated measures ANOVA with Bonferroni post-hoc test. Correlation of two variants was analyzed by Pearson correlation coefficient. Patient survival rate was analyzed using the Kaplan-Meier method followed by log-rank test. A *p* < 0.05 value was considered to be indicative of statistical significance.

## Results

### miR-216b is poorly-expressed in OS and its high expression is positively-correlated with patient survival

**Fig. 1:**
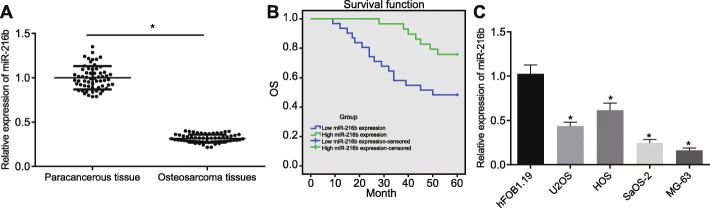
miR-216b expression is decreased in OS and its high expression is positively correlated with patient survival. **a** miR-216b expression was determined by RT-qPCR in OS and paracancerous tissues from patients (*n* = 60), relative to U6. * *p* < 0.05 vs. paracancerous tissues by paired *t*-test. **b** Overall survival in OS patients was determined by the Kaplan-Meier method. **c** miR-216b expression was determined by RT-qPCR in OS cell lines and normal hFOB1.19 cell line, relative to U6. * *p* < 0.05 vs. hFOB1.19 cell line by one-way ANOVA, followed by Tukey’s test. Data in panel (**b**) were compared with unpaired *t*-test. Data are shown as mean ± standard deviation of three technical replicates

Firstly, the expression of miR-216b was found to be markedly lower in OS tissues compared to that in paracancerous tissues (fold = 0.315) (Fig. 1a). The median miR-216b relative expression pattern in cancer tissues from 60 patients was subsequently determined, after which the patients with values above the median were placed into the high miR-216b expression group, while those below the median were regarded as the low miR-216b expression group. In addition, Kaplan-Meier survival analysis revealed that elevated expression of miR-216b was correlated with higher overall survival in OS patients (Fig. 1b). However, no significant correlation was identified between the miR-216b expression and factors such as age, sex, location of the tumor, and metastasis (Table 2). Additionally, results of multivariate analysis demonstrated that miR-216b served as an independent prognostic factor for OS (Table 3). Lastly, the expression of miR-216b was determined to be significantly lower in the MG63 and SaOS-2 cell lines relative to other OS cell lines and control (hFOB1.19 cell line) (U2OS: fold = 0.436, HOS: fold = 0.614, SaOS-2: fold = 0.248, MG63: fold = 0.161) (Fig. 1c). Hence, MG63 and SaOS-2 cells were selected for subsequent experimentation.

**Table 2:** Relationship between clinical characteristics in OS patients and low or high miR-261b expression (*n* = 60)

Demographics	n	miR-261b expression (%)		*P*
		Low (*n* = 31)	High (*n* = 29)	
Sex				0.456
Male	40	22	18	
Female	20	9	11	
Age (years)				0.317
≤ 20	37	21	16	
> 20	23	10	13	
Location				
Femur	39	24	20	0.459
Others	21	7	9	
Postoperative recurrence				0.001^*^
Presence	44	20	6	
Absence	16	11	23	
Distant metastasis				0.381
Presence	22	13	9	
Absence	38	18	20	

* indicates significant difference by chi-square test

**Table 3:** Univariate and multivariate analyses for predictors of overall survival in OS patients

Variable	Univariate Analysis		Multivariate Analysis	
	HR (95% CI)	*p*	HR (95% CI)	*p*
Gender	0.769 (0.316–2.81)	0.562	0.804 (0.285–2.270)	0.681
Age	0.800 (0.339–1.887)	0.611	0.943 (0.355–2.507)	0.907
Location	0.673 (0.250–1.814)	0.434	0.773 (0.274–2.186)	0.628
Distant metastasis	0.652 (0.286–1.488)	0.310	0.717 (0.297–1.731)	0.459
Histologic grade	1.348 (0.594–3.056)	0.475	1.052 (0.394–2.806)	0.920
Enneking grade	1.270 (0.557–2.898)	0.570	1.124 (0.477–2.651)	0.789
miR-216b expression	0.338 (0.139–0.823)	0.017^*^	0.363 (0.142–0.931)	0.035^*^

* indicates significant difference

### miR-216b improves the effects of cisplatin on OS cells in vitro

**Fig. 2:**
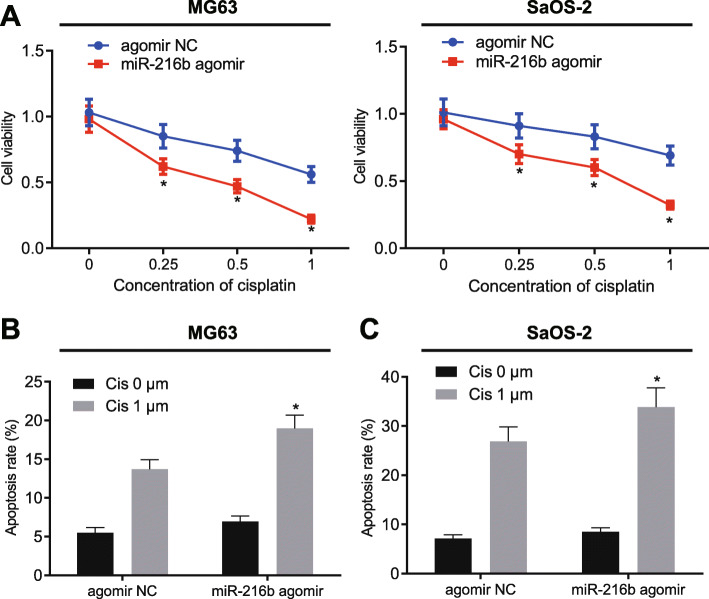
miR-216b enhances cisplatin-induced apoptosis in OS cells. **a** Cell viability in MG63 and SaOS-2 cells was determined by CCK-8 assay upon treatment with miR-216b agomir. **b** Apoptosis of MG63 cells was determined by flow cytometry upon treatment with miR-216b agomir. **c** Apoptosis of SaOS-2 cells was determined by flow cytometry upon treatment with miR-216b agomir. * *p* < 0.05 vs. cells treated with agomir NC. Data in panel (**a**) and (**c**) were compared by unpaired t-test while data in panel (**b**) were compared by repeated measures ANOVA with Bonferroni test. Data are shown as mean ± standard deviation of three technical replicates

We subsequently set out to evaluate the sensitivity of MG63 and SaOS-2 cells to cisplatin as OS resistance against chemotherapy remains a significant obstacle during the treatment of OS. Transfection of miR-216b agomir, as expected, elevated the expression of miR-216b in MG63 and SaOS-2 cells, indicating successful transfection (MG63: fold = 2.218, SaOS-2: fold = 3.094) (Supplementary Fig. 1). Meanwhile, it was found that cisplatin decreased the cell viability in MG-63 and SaOS-2 cells but much more so in cells transfected with miR-216b agomir (MG63: 0.25 fold = 0.73, 0.5 fold = 0.64, 1 fold = 0.39; SaOS-2: 0.25 fold = 0.77, 0.5 fold = 0.72, 1 fold = 0.46) (Fig. 2a). Cisplatin was found to also augment cell apoptosis, which was further elevated following miR-216b agomir treatment in cells (MG63: miR-216b agomir fold = 1.39; SaOS-2: miR-216b agomir fold = 1.26) (Fig. 2b, c). These results suggested that miR-216b enhanced the promotive effects of cisplatin on OS cell apoptosis, while inhibiting cell viability in vitro.

### miR-216b targets and inhibits JMJD2C to enhance cisplatin-induced apoptosis in OS cells

**Fig. 3:**
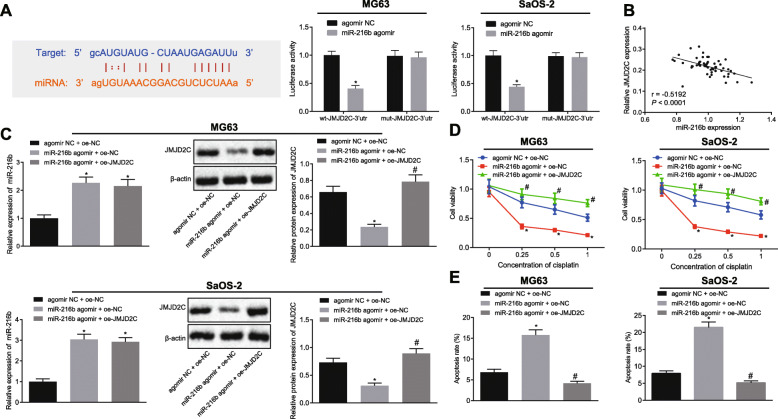
miR-216b targets JMJD2C and inhibits its expression to enhance cisplatin-induced apoptosis in vitro. **a** miR-216b binding to JMJD2C was determined by the bioinformatics website Starbase in combination with the dual luciferase reporter assay in cells. agomir NC, miR-216b agomir, wt-JMJD2C-3’UTR and mut-JMJD2C-3’UTR were co-transfected into MG63 and SaOS-2 cells and then the luminescence intensity was determined. **b** Pearson correlation of miR-216b expression with JMJD2C expression in OS tissues (*n* = 60). **c** miR-216b expression and JMJD2C mRNA expression were determined by RT-qPCR in cells, relative to U6 and β-actin, respectively, and representative Western blots of JMJD2C protein and its quantitation in cells, relative to β-actin. **d** Cell viability in MG63 and SaOS-2 cells was determined by CCK-8 assay. **e** Apoptosis of MG63 and SaOS-2 cells was determined by flow cytometry. * *p* < 0.05 vs. cells treated with agomir NC + oe-NC, # *p* < 0.05 vs. cells treated with miR-216b agomir + oe-NC. Data in panel (**a**) were compared by unpaired *t*-test, in panel (**c**) and (**e**) using one-way ANOVA, with Tukey’s test and in panel (**b**) by repeated measures ANOVA with Bonferroni test. Data are shown as mean ± standard deviation of three technical replicates

The online bioinformatic website Starbase [36] predicted the presence of binding sites of miR-216b to the 3’UTR of the histone demethylase JMJD2C mRNA (KDM4C). Subsequently, a dual luciferase reporter assay was performed, which revealed that co-transfection of miR-216b agomir with wt-JMJD2C-3’UTR triggered a reduction in the luminescence intensity (MG63 fold = 0.407; SaOS-2 fold = 0.442), whereas no alterations were detected upon co-transfection with mut-JMJD2C-3’UTR in cells (MG63 fold = 0.974; SaOS-2 fold = 0.981) (Fig. 3a). Furthermore, Pearson correlation analysis highlighted a negative correlation between the expression of miR-216b and JMJD2C in OS tissues (Fig. 3b). In addition, miR-216b agomir was identified to increase the expression of miR-216b, while reducing the mRNA and protein expression of JMJD2C in MG-63 and SaOS-2 cells (MG63-RT-qPCR miR-216b agomir + oe-NC fold = 2.263; miR-216b agomir + oe-JMJD2C fold = 2.155) (SaOS-2-RT-qPCR miR-216b agomir + oe-NC fold = 3.043, miR-216b agomir + oe-JMJD2C fold = 2.955) (MG63-western blot analysis miR-216b agomir + oe-NC fold = 0.359; miR-216b agomir + oe-JMJD2C fold = 3.316) (SaOS-2-western blot analysis miR-216b agomir + oe-NC fold = 0.426; miR-216b agomir + oe-JMJD2C fold = 2.862) (Fig. 3c). It was further found that miR-216b diminished the cisplatin-induced decrease in cell viability (MG63 0.25 fold = 0.47, 0.5 fold = 0.45, 1 fold = 0.41) (SaOS-2 0.25 fold = 0.45, 0.5 fold = 0.41, 1 fold = 0.37), the effect of which was weakened following JMJD2C over-expression (MG63 0.25 fold = 2.55, 0.5 fold = 2.80, 1 fold = 3.68) (SaOS-2 0.25 fold = 2.71, 0.5 fold = 3.24, 1 fold = 3.81) (Fig. 3d). Similarly, miR-216b was observed to enhance cisplatin-induced apoptosis (MG63 fold = 2.32; SaOS-2 fold = 2.73), the effect of which was weakened by JMJD2C over-expression (MG63 fold = 0.26; SaOS-2 fold = 0.24) (Fig. 3e). Based on these results, it could be inferred that miR-216b could bind to JMJD2C and inhibit its expressions, ultimately enhancing cisplatin-induced apoptosis in OS cells.

### JMJD2C enhances HES1 expression by interacting with HIF1α to remove H3K9 methylation modification at the HES1 promoter region in vitro

**Fig. 4:**
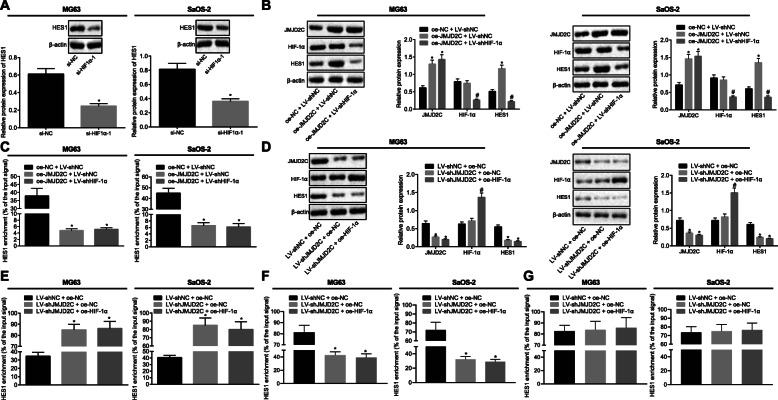
JMJD2C enhances HES1 expression by interacting with HIF1α to remove H3K9 methylation modification at the HES1 promoter region in cells. **a** Representative Western blots of HES1 protein and its quantitation in MG-63 and SaOS-2 cells treated with si-HIF1α, relative to β-actin. * *p* < 0.05 vs. cells treated with si-NC by unpaired *t*-test. **b** Representative Western blots of JMJD2C, HIF1α and HES1 proteins and their quantitation in MG-63 and SaOS-2 cells treated with si-HIF1α, relative to β-actin. * *p* < 0.05 vs. cells treated with oe-NC + LV-shNC. **c** The quantity of the HES1 promoter in the immune complexes was determined by ChIP-qPCR assay in MG-63 and SaOS-2 cells treated with oe-JMJD2C or in combination with shHIF1α. * *p* < 0.05 vs. cells treated with oe-NC + LV-shNC. **d** Representative Western blots of JMJD2C, HIF1α, and HES1 proteins and their quantitation in MG-63 and SaOS-2 cells treated with oe-JMJD2C or in combination with shHIF1α, relative to β-actin. * *p* < 0.05 vs. cells treated with LV-shNC + oe-NC. **e** The quantity of the HES1 promoter in the immune complexes was determined by ChIP-qPCR assay in MG-63 and SaOS-2 cells treated with shJMJD2C or in combination with oe-HIF1α. **f**, **g** The quantity of HES1 promoter in the immune complexes with anti-H3K9me2 (**f**) and anti-H3 (**g**) in MG-63 and SaOS-2 cells treated with shJMJD2C or in combination with oe-HIF1α. * *p* < 0.05 vs. cells treated with LV-shNC + oe-NC. Data in panel (**c**-**h**) were performed using one-way ANOVA with Tukey’s test. Data are shown as mean ± standard deviation of three technical replicates

In order to characterize the effect of JMJD2C/HIF1α on HES1, we silenced HIF1α in cells, and subsequently identified that si-HIF1α-3 exhibited the best HIF1α silencing efficiency (MG63 si-HIF1α-1 fold = 0.504, si-HIF1α-2 fold = 0.401, si-HIF1α-3 fold = 0.328) (SaOS-2 si-HIF1α-1 fold = 0.512, si-HIF1α-2 fold = 0.392, si-HIF1α-3 fold = 0.250), and was thus chosen for the following experiments (Supplementary Fig. 2). HIF1α silencing was found to down-regulate the expression of HES1 in MG-63 and SaOS-2 cells (MG63 fold = 0.403, SaOS-2 fold = 0.445) (Fig. 4a). HIF1α silencing also reversed the elevation in HES1 expression caused by JMJD2C over-expression, demonstrating that HIF1α functioned as a crucial factor in the up-regulation of HES1 by JMJD2C and HIF1α (MG63 fold = 2.287, SaOS-2 fold = 2.260) (Fig. 4b). ChIP-qPCR assay was then employed to detect the quantity of the HES1 promoter in the immune complexes in MG-63 and SaOS-2 cells, the results of which revealed reduced amounts of HES1 promoter in the immune complexes upon JMJD2C over-expression (MG63 fold = 0.13, SaOS-2 fold = 0.15), while no significant differences were found regarding the quantity of the HES1 promoter (MG63 fold = 1.08, SaOS-2 fold = 0.93) following combined treatment with oe-JMJD2C and shHIF1α(Fig. 4c). Meanwhile, HIF1α over-expression failed to reverse the down-regulation of HES1 brought about by JMJD2C silencing (MG63-LV-shJMJD2C + oe-NC JMJD2C fold = 0.416, HIF1α fold = 1.144, HES1 fold = 0.325) (MG63-LV-shJMJD2C + oe-HIF1α JMJD2C fold = 0.742, HIF1α fold = 1.908, HES1 fold = 0.820) (SaOS-2-LV-shJMJD2C + oe-NC JMJD2C fold = 0.496, HIF1α fold = 1.135, HES1 fold = 0.396) (SaOS-2-LV-shJMJD2C + oe-HIF1α JMJD2C fold = 0.833, HIF1α fold = 1.832, HES1 fold = 0.831) (Fig. 4d). These results demonstrated that JMJD2C and HIF1α were both essential in the up-regulation of HES1. ChIP-qPCR assay was further applied to detect the quantity of the HES1 promoter enriched by IgG and H3K9me3 antibodies in MG-63 and SaOS-2 cells, and the results demonstrated that HIF1α over-expression (MG63 fold = 1.01, SaOS-2 fold = 0.94) exhibited no effect on the elevated H3K9me3 levels caused by JMJD2C silencing (MG63 fold = 2.61, SaOS-2 fold = 2.37) (Fig. 4e). In addition, the quantity of HES1 promoter in the immune complexes with anti-H3K9me2 and anti-H3 antibodies was determined in MG-63 and SaOS-2 cells following different treatments (Fig. 4f-g). The quantity of HES1 promoter in the immune complexes with anti-H3K9me2 was found to be diminished (MG63 fold = 0.52, SaOS-2 fold = 0.45) in response to JMJD2C silencing, whereas combined treatment with JMJD2C silencing and HIF1α over-expression did not negate the effect of JMJD2C silencing (MG63 fold = 0.92, SaOS-2 fold = 0.89) (Fig. 4f). Besides, JMJD2C silencing and HIF1α over-expression alone or in combination did not affect the quantity of HES1 promoter in the immune complexes with anti-H3 (MG63 fold = 1.02, SaOS-2 fold = 1.04) (Fig. 4g). These findings highlighted the ability of JMJD2C to up-regulate HES1 expression via HIF1α interaction to diminish H3K9 methylation modification in vitro.

### miR-216b enhances cisplatin-induced apoptosis via regulation of the JMJD2C/HIF1α/HES1 signaling axis in OS cells

**Fig. 5:**
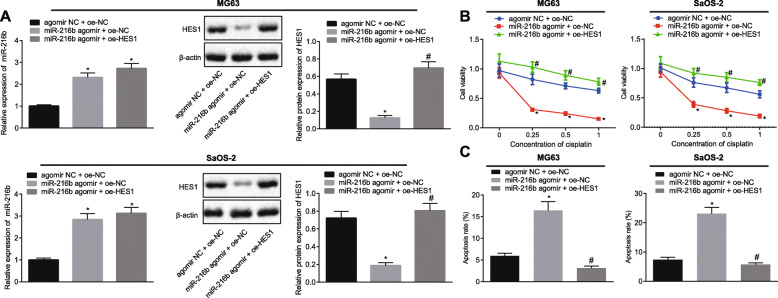
The miR-216b/JMJD2C/HIF1α/HES1 signaling axis is involved in cisplatin-induced apoptosis in OS cells. **a** Transfection efficiency of miR-216b and HES1 expressions in MG63 and SaOS-2 cells was determined by RT-qPCR and Western blot analysis, relative to U6 and β-actin, respectively. **b** Cell viability of MG63 and SaOS-2 cells was determined by CCK-8 assay. **c** Apoptosis of MG63 and SaOS-2 cells was determined by flow cytometry. * *p* < 0.05 vs. cells treated with agomir NC + oe-NC; # *p* < 0.05 vs. cells treated with miR-216b agomir + oe-NC. Data in panel (**a**) and (**c**) were compared by one-way ANOVA with Tukey’s test, while data in panel (**b**) were compared by repeated measures ANOVA with Bonferroni test. Data are shown as mean ± standard deviation of three technical replicates

The transfection of miR-216b agomir and oe-HES1 in MG-63 and SaOS-2 cells was efficient for further experimentation (MG63-RT-qPCR miR-216b agomir + oe-NC fold = 2.315, miR-216b agomir + oe-HES1 fold = 2.721) (SaOS-2-RT-qPCR miR-216b agomir + oe-NC fold = 2.845, miR-216b agomir + oe-HES1 fold = 3.126) (MG63-western blot analysis miR-216b agomir + oe-NC fold = 0.222, miR-216b agomir + oe-HES1 fold = 5.532) (SaOS-2-western blot analysis miR-216b agomir + oe-NC fold = 0.259, miR-216b agomir + oe-HES1 fold = 4.310) (Fig. 5a). Over-expression of miR-216b was found to diminish the cisplatin-induced reduction in the viability in MG-63 and SaOS-2 cells (MG63 0.25 fold = 0.38, 0.5 fold = 0.34, 1 fold = 0.24), (SaOS-2 0.25 fold = 0.51, 0.5 fold = 0.42, 1 fold = 0.33), the effect of which was abrogated by HES1 over-expression (MG63 0.25 fold = 2.36, 0.5 fold = 3.04, 1 fold = 4.02) (SaOS-2 0.25 fold = 3.32, 0.5 fold = 3.71, 1 fold = 5.20) (Fig. 5b). In addition, miR-216b over-expression further enhanced the cisplatin-induced apoptosis in MG-63 and SaOS-2 cells (MG63 fold = 2.76, SaOS-2 fold = 3.12), whereas HES1 over-expression brought about the opposite effects (MG63 fold = 0.19, SaOS-2 fold = 0.25) (Fig. 5c). These results in concert with those discussed above, demonstrated the effect of miR-216b/JMJD2C/HIF1α/HES1 signaling axis on enhancing the effects of chemotherapy in OS cells.

### miR-216b enhances the effects of cisplatin via regulation of the JMJD2C/HIF1α/HES1 signaling axis in tumor xenografts of human OS cells

**Fig. 6:**
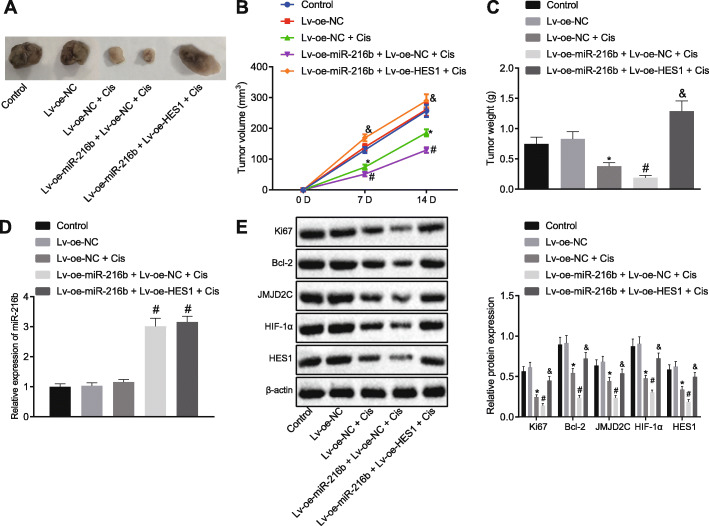
The miR-216b/JMJD2C/HIF1α/HES1 signaling axis participates in cisplatin-induced apoptosis in vivo. **a** Representative images of transplanted tumors from nude mice. **b** The growth of OS xenograft tumor in nude mice was measured every 7 days. **c** Tumor weight in nude mice. **d** miR-216b expression was determined by RT-qPCR in tumor tissues of nude mice, relative to U6. **e** Representative Western blots of Ki67, Bcl-2, JMJD2C, HIF1α and HES1 proteins and their quantitation in tumor tissues, relative to β-actin. * *p* < 0.05 vs. mice treated with Lv-oe-NC; # *p* < 0.05 vs. mice treated with Lv-oe-NC + Cis; & *p* < 0.05 vs. mice treated with Lv-oe-miR-216b + Lv-oe-NC + Cis. Data are expressed as mean ± standard deviation. Data in panel (**a**) and (**c**-**e**) were compared using one-way ANOVA with Tukey’s test, while data in panel (**b**) were compared by repeated measures ANOVA with Bonferroni test. *n* = 10 for mice upon each treatment

Representative tumor xenografts isolated from nude mice are depicted in Fig. 6a. Tumor growth (day 7 fold = 0.53, day 14 fold = 0.71) (Fig. 6b) and weight (fold = 0.46) (Fig. 6c) were found to be reduced by cisplatin, while this effect was further increased upon miR-216b over-expression (tumor growth: day 7 fold = 0.69, day 14 fold = 0.70) (tumor weight: fold = 0.51). Meanwhile, the addition of a HES1 over-expression plasmid weakened the effect of miR-216 over-expression on tumor growth (day 7 fold = 3.30, day 14 fold = 2.24) and weight (fold = 6.70). Moreover, in tumor tissues, miR-216b expression was observed to be increased, and further increased following miR-216b over-expression. However, no changes were observed in miR-216b expression following the simultaneous over-expression of miR-216b and HES1 in tumor tissues (Fig. 6d). Furthermore, cisplatin was found to diminish the protein expressions of Ki67, Bcl-2, JMJD2C, HIF1α and HES1 (Ki67 fold = 0.399, Bcl-2 fold = 0.593, JMJD2C fold = 0.649, HIF1α fold = 0.529, HES1 fold = 0.547), all of which were further down-regulated by miR-216b over-expression (Ki67 fold = 0.581, Bcl-2 fold = 0.437, JMJD2C fold = 0.535, HIF1α fold = 0.640, HES1 fold = 0.548) (Fig. 6e). HES1 over-expression was also identified to weaken the effects associated with miR-216b over-expression (Ki67 fold = 3.172, Bcl-2 fold = 3.051, JMJD2C fold = 2.274, HIF1α fold = 2.373, HES1 fold = 2.662). These in vivo results provided evidence confirming the promotive role of miR-216b on the effects of cisplatin via the JMJD2C/HIF1α/HES1 signaling axis.

## Discussion

Although not as prevalent as many other malignancies, OS represents the foremost primary bone cancer with the vast majority of cases seen among younger populations [1]. In addition, the 5-year survival rate worldwide has failed to improve for decades, remaining between 40 and 70% [3–5]. This can be partly attributed to the emergence of chemotherapeutic resistance in the treatment of OS [37, 38]. Meanwhile, the aberrant regulatory interactions between miRNA-mRNA can potentially mediate the malignant phenotypes of various cancer cells, some of which have shown promise as potential novel targets and therapies aimed at limiting the angiogenesis of malignancies, including OS [39]. Thus, identification of novel non-invasive biomarkers with miRNA-mRNA interaction for the prevention and treatment of OS might prove highly-beneficial. Findings obtained in the current study indicated that miR-216b could augment the effect of the chemotherapeutic agent cisplatin on cell viability and apoptosis in OS cells, which might be attributed to its binding to the JMJD2C gene and the subsequent inhibition of JMJD2C expression, leading to the regulation of the HIF1α/HES1 signaling axis. Overall, our study demonstrated the role of the JMJD2C/HIF1α/HES1 signaling axis in the miR-216b-mediated enhancement of the anti-cancer effects of cisplatin.

Firstly, during the course of the current study, we identified that the expression of miR-216b was markedly reduced in OS, whereas a positive correlation was detected between high expression levels of miR-216b and patient survival. Furthermore, our findings revealed that miR-216b could heighten the effect of cisplatin, resulting in reduced OS cell viability and elevated cell apoptosis. Existing literature has demonstrated that miR-216b possesses the ability to directly reduce cancer cell proliferation, migration, and invasion in OS [13]. In addition, another study proposed the use of miR-216b as a potential sensitizer in cisplatin chemotherapy owing to its ability to reduce cell viability and promote the apoptosis of cisplatin-resistant ovarian cancer cells [40]. Diminished levels of miR-216b have been detected in non-small cell lung cancer cells while enhanced miR-216b expression has been demonstrated to elevate cisplatin-induced apoptosis by targeting Beclin-1 [41]. All in all, these findings and evidence suggest that miR-216b could serve as a promising target to enhance the effects of cisplatin on OS cells.

Moreover, miRNAs are known to possess the capacity to modulate gene expressions at a post-transcriptional level by means of interacting with the 3’UTR of specific target mRNAs [42]. During the current study, online biological prediction and luciferase reporter assay were employed, which revealed data indicating that miR-216b could bind to the 3’UTR of the JMJD2C mRNA and negatively-regulate its expression. In line with our findings, JMJD2C has been previously reported to be highly-expressed in OS, and even capable of elevating the aggressiveness of OS [17]. Therefore, it would be reasonable to speculate that miR-216b could elicit an inhibitory effect on the JMJD2C expression, consequently delaying the progression of OS. Moreover, recent data have evidenced that knockdown of JMJD2A decreases the proliferation of ovarian cancer cells, while simultaneously acting to improve the sensitivity of ovarian cancer cells to cisplatin [43]. The aforementioned findings support the notion that miR-216b binds to JMJD2C and subsequently inhibits its expression, ultimately enhancing cisplatin-induced apoptosis of OS cells.

Furthermore, our findings revealed that JMJD2C up-regulated the expression of the HES1 gene via HIF1α interaction to diminish H3K9 methylation modification. Several studies have stated that HIF1α is an essential component of JMJD2C functioning [18, 20]. Moreover, HIF1α and HES1 have been reported to work in tandem to decrease the effects of cisplatin on OS cells, which is very much in line with our findings [22]. As a result, we highlighted JMJD2C and HIF1α as downstream signaling molecules of miR-216b, wherein miR-216b reduced JMJD2C expression, likely interfering with mRNA stability and JMJD2C action in the demethylation of HES1 gene at the promoter region. More importantly, high expression levels of HES1 have been previously correlated with enhanced OS cell proliferation, migration and invasion as well as boosted chemoresistance [44]. What’s more, our findings revealed that miR-216b diminished the expression of HES1 via the removal of H3K9 methylation at the gene promoter site of HES1 in OS cells, which has been previously emphasized as a critical factor for HES1 activation [27]. In contrast, HES1 over-expression weakened the effects of miR-126b, as evidenced by suppressed tumor growth and enhanced cell apoptosis in OS. These results are largely consistent with the previous evidence indicating that HES1 promotes chemotherapy resistance in different cancers [25, 26]. In addition, we uncovered that HES1 over-expression brought about elevations in the expression of Ki67 and Bcl-2. This is particularly important as Ki67 is recognized as a marker of cancer cell proliferation in OS [45]. Similarly, Bcl-2 is well-documented as a suppressor of apoptosis in various cancers including OS [46]. Consequently, we are convinced that miR-216b may have the potential to enhance cisplatin-induced apoptosis via regulation of the JMJD2C/HIF1α/HES1 signaling axis in OS.

## Conclusion

Overall, the current study highlighted that miR-216b augmented cisplatin-induced apoptosis through regulation of the JMJD2C/HIF1α/HES1 signaling axis. The newly found miR-216b/JMJD2C/HIF1α/HES1 signaling axis might pave the way for potential therapeutic mechanisms to reduce chemoresistance in OS. Nevertheless, a few OS cell lines were initially screened in the study, but only MG-63 and SaOS-2 cells were chosen due to highest expression profiles of miR-216b. This selection criterion may pose a selection bias. Therefore, results from our study should be confirmed by future studies incorporating other types of OS cell lines. The current study lends support to the notion that miR-216b may be employed as a chemotherapy adjunct alongside cisplatin for the efficacious treatment of OS.

## Supplementary information

**Additional file 1: Supplementary Fig. 1.** miR-216b transfection efficiency was determined by RT-qPCR in MG63 and SaOS-2 cells. * *p* < 0.05 vs. cells treated with agomir NC by unpaired t-test. Data are shown as mean ± standard deviation of three technical replicates.

**Additional file 2: Supplementary Fig. 2.** Silencing efficiency of HIF1α was determined by RT-qPCR in MG-63 and SaOS-2 cells. * *p* < 0.05 vs. cells treated with si-NC by unpaired *t*-test.

## Data Availability

The datasets generated/analyzed during the current study are available.

## Abbreviations

OS: Osteosarcoma
miRs or miRNAs: microRNAs
JMJD2C: Jumonji C domain-containing 2C
oe: Overexpression
LV: Lentivirus
sh: Short hairpin RNA
NC: Negative control
HIF1α: Hypoxia-inducible factor 1
HES1: Hairy and enhancer of split-1
Co-IP: Co-immunoprecipitation
ChIP: Chromatin immunoprecipitation
DMEM: Dulbecco’s modified Eagle’s medium
NCBI: National Center for Biotechnology Information
wt: Wild type
mut: Mutant
3’UTR: 3’untranslated region
cDNA: Complementary DNA
RT-qPCR: Reverse transcription quantitative polymerase chain reaction
RIPA: Radio-immunoprecipitation assay
PMSF: Phenylmethanesulfonyl fluoride
BCA: Bicinchoninic acid
SDA-PAGE: Sodium dodecyl sulfate polyacrylamide gel electrophoresis
PVDF: Polyvinylidene fluoride
IgG: Immunoglobulin G
TBST: Tris-buffered saline with Tween-20
HRP: Horseradish peroxidase
ECL: Enhanced chemiluminescence
Bcl-2: B-cell lymphoma 2
CCK-8: Cell counting kit-8
FITC/PI: Fluorescein isothiocyanate/propidium iodide
ANOVA: Analysis of variance
