# Correction to: microRNA-216b enhances cisplatin-induced apoptosis in osteosarcoma MG63 and SaOS-2 cells by binding to JMJD2C and regulating the HIF1α/HES1 signaling axis

**DOI:** 10.1186/s13046-020-01804-7

**Published:** 2021-01-07

**Authors:** Dong Yang, Tianyang Xu, Lin Fan, Kaiyuan Liu, Guodong Li

**Affiliations:** Department of Orthopedics, Shanghai Tenth People’s Hospital, Tongji University School of Medicine, Shanghai, 200433 P.R. China

**Correction to: J Exp Clin Cancer Res 39, 201 (2020)**

**https://doi.org/10.1186/s13046-020-01670-3**

Following publication of the original article [1], the authors identified an error in the author affiliation. The current author affiliation is not complete.

The complete author affiliation is: Department of Orthopedics, Shanghai Tenth People’s Hospital, Tongji University School of Medicine, Shanghai, 200433, P.R. China.

The original article has been updated.
